# Investigating the association between schizophrenia and distance visual acuity: Mendelian randomisation study

**DOI:** 10.1192/bjo.2023.6

**Published:** 2023-02-07

**Authors:** Natalie Shoham, Diana Dunca, Claudia Cooper, Joseph F. Hayes, Andrew McQuillin, Nick Bass, Gemma Lewis, Karoline Kuchenbaecker

**Affiliations:** Division of Psychiatry, University College London, UK; and Islington Early Intervention Service, Camden and Islington NHS Foundation Trust, St Pancras Hospital, London, UK; UCL Genetics Institute, University College London, UK; Centre for Psychiatry and Mental Health, Wolfson Institute of Population Health, Queen Mary University of London, UK; and Tower Hamlets Memory Service, East London NHS Foundation Trust, London, UK; Division of Psychiatry, University College London, UK; and Camden and Islington NHS Foundation Trust, St Pancras Hospital, London, UK; Division of Psychiatry, University College London, UK; Division of Psychiatry, University College London, UK; and Tower Hamlets Memory Service, East London NHS Foundation Trust, London, UK; Division of Psychiatry, University College London, UK; and UCL Genetics Institute, University College London, UK

**Keywords:** Psychiatric epidemiology, visual impairment, schizophrenia, psychosis, Mendelian randomisation

## Abstract

**Background:**

Increased rates of visual impairment are observed in people with schizophrenia.

**Aims:**

We assessed whether genetically predicted poor distance acuity is causally associated with schizophrenia, and whether genetically predicted schizophrenia is causally associated with poorer visual acuity.

**Method:**

We used bidirectional, two-sample Mendelian randomisation to assess the effect of poor distance acuity on schizophrenia risk, poorer visual acuity on schizophrenia risk and schizophrenia on visual acuity, in European and East Asian ancestry samples ranging from approximately 14 000 to 500 000 participants. Genetic instrumental variables were obtained from the largest available summary statistics: for schizophrenia, from the Psychiatric Genomics Consortium; for visual acuity, from the UK Biobank; and for poor distance acuity, from a meta-analysis of case–control samples. We used the inverse variance-weighted method and sensitivity analyses to test validity of results.

**Results:**

We found little evidence that poor distance acuity was causally associated with schizophrenia (odds ratio 1.00, 95% CI 0.91–1.10). Genetically predicted schizophrenia was associated with poorer visual acuity (mean difference in logMAR score: 0.024, 95% CI 0.014–0.033) in European ancestry samples, with a similar but less precise effect that in smaller East Asian ancestry samples (mean difference: 0.186, 95% CI –0.008 to 0.379).

**Conclusions:**

Genetic evidence supports schizophrenia being a causal risk factor for poorer visual acuity, but not the converse. This highlights the importance of visual care for people with psychosis and refutes previous hypotheses that visual impairment is a potential target for prevention of schizophrenia.

## Schizophrenia

Multiple studies have found evidence of a cross-sectional association between psychotic illnesses and visual impairment, but the direction of association is unclear.^1–4^ Schizophrenia causes marked detriment to quality of life and life expectancy.^5,6^ Existing treatments do not ameliorate these effects,^7^ and prevention would be preferable because of the considerable distress and social, mental and cognitive harms experienced.

## Myopia/poor distance acuity

Myopia, or short-sightedness, is a type of refractive error. It is the most common cause of distance visual impairment globally, alongside cataracts.^8^ It occurs when light entering the eye falls short of the retina, creating blurred images at distance (poorer visual acuity).^9^ Prevalence is increasing, and myopia is predicted to affect 50% of the global population by 2050.^10^ In some populations in East Asia, 80–90% of school-leavers are already affected.^11^ Myopia can usually be corrected by visual aids, but this typically relies on the initiative of individuals to seek optical care, which is often only available privately.^9^

## Visual impairment and psychosis

A meta-analysis of cross-sectional studies investigating the association between visual acuity impairment and psychosis, which cannot elucidate the direction of effect, found an association (odds ratio 1.76, 95% CI 1.34–2.31) in studies classing visual acuity impairment of any cause and psychosis (odds ratio 1.85, 95% CI 1.17–2.92) as the exposure.^4^

Several studies have considered whether visual impairment could be a causal risk factor for psychosis.^12^ Theoretical rationale for this is described in the Protection against Schizophrenia (PaSZ) model.^13^ This model highlights the absence of reported cases of a person with congenital cortical blindness developing schizophrenia. It proposes that congenitally blind individuals are protected from aberrant visual input – a risk factor for schizophrenia.^13^ It has been noted that cognitive alterations, such as improved working memory, which enable congenitally blind people to perceive the world without vision are the reverse of typical deficits in schizophrenia, perhaps creating a buffering effect.^14^ Conversely, individuals who lose vision but lack these adaptations might have elevated risk because of a reliance on visual input to process surroundings.^14^ Two large longitudinal studies of children and young adults found that myopia is associated with future psychosis.^12,15^

Conversely, psychotic illnesses could be a causal risk factor for visual impairment. Affected individuals may experience ocular side-effects from antipsychotic medications and are at increased risk of comorbidities that impair eyesight.^1,16,17^ They may also spend more time indoors, a risk factor for myopia.^18^ N-methyl-D-aspartate (NDMA) receptor dysfunction has also been implicated in both impaired visual acuity and schizophrenia.^19,20^ Several cross-sectional studies have reported higher rates of myopia and lower self-reported recent optician attendance in people with schizophrenia and other psychotic illnesses than in the general population.^1,21–23^ To our knowledge, this association has not previously been explored in longitudinal studies.

A third possible explanation for the association between visual impairment and psychosis is that these conditions share an underlying neurological vulnerability, or that there is confounding by other variables such as socioeconomic status and general health. The possibility of shared neurological vulnerability is supported by functional and imaging studies showing retinal changes in people with psychotic illnesses and their offspring.^24–26^

## Mendelian randomisation

Identifying the nature of the association could aid prevention or treatment of either condition. Observational cohort studies are subject to unmeasured and residual confounding, and cannot completely exclude reverse causation. Mendelian randomisation aims to measure causal relationships by minimising the effects of confounding and reverse causation.^10^ It uses genetic variants as proxy exposures to simulate a randomised design, since genetic variants were randomly allocated at conception.^27^ Assumptions of Mendelian randomisation methodology include relevance (that genetic variants associate with the exposure), independence (that they share no common cause with outcome) and exclusion restriction (that they influence the outcome exclusively via the exposure).^27^ We aimed to conduct the first Mendelian randomisation study to test the hypotheses that poorer distance visual acuity is a causal risk factor for schizophrenia, and schizophrenia is a causal risk factor for poorer distance visual acuity.

## Method

We followed the Strengthening the Reporting of Observational Studies in Epidemiology using Mendelian Randomization (STROBE-MR) guidelines when reporting results.^28^ No individual-level data was used in this study; therefore participant consent was not required.

We used summary statistics (published effect sizes and standard errors from genome-wide association studies (GWAS)) to conduct bidirectional, two-sample Mendelian randomisation. Two-sample Mendelian randomisation allows for the largest samples to be employed in finding genetic instruments, regardless of whether the instruments’ association with the outcome was measured in the same sample, increasing power.^29^

We ran a comparison analysis using dental caries in place of poor distance acuity where an association was found, to assess neglect of healthcare as a possible mechanism.

### Samples used in schizophrenia GWAS

#### Psychiatric Genomics Consortium

The Psychiatric Genomics Consortium (PGC) is a consortium of case–control studies aimed at identifying genetic variants associated with psychiatric disorders.^7^ The data-set comprises 90 studies, including 67 390 schizophrenia cases and 94 015 controls, of which 80% had European ancestry (*N* = 129 124).^7^ Across samples, cases could be defined as follows: diagnosis of schizophrenia, schizoaffective disorder or schizophrenia spectrum disorder determined through consensus between psychiatrists; validated diagnostic interview; structured assessment; review of medical records or a combination of these.^7^ Analysed separately were 22 778 schizophrenia cases and 35 362 controls of East Asian ancestry (*N* = 58 140).^30^

### Samples used in myopia, refractive error and visual acuity GWAS

#### UK Biobank

Between 2006 and 2010, over 500 000 UK residents aged 40–60 years were recruited to the UK Biobank cohort.^31^ Further details are available.^32^ A range of health variables are assessed by questionnaires, examination and blood sampling in this ongoing, longitudinal study. Strength of lens required for correction of refractive error, or spherical equivalent, is measured in dioptres. Myopia status was determined either by spherical equivalent measured by autorefractor, or inferred using a questionnaire and other data: age, gender, age of first spectacle or contact lens wear, and year of birth.^33^ A total of 102 117 participants had both measured refractive error and genotyping.^33^ An additional 108 956 cases and 70 941 controls had inferred myopia status.^33^ Myopia status was meta-analysed,^33^ and contributed to our poor distance acuity exposure instrument.

We also used UK Biobank summary statistics for the phenotype of continuous visual acuity. At baseline assessment, 116 011 participants had their habitual visual acuity measured, using their usual corrective aids.^34^ Of these, 90 214 had European ancestry and 923 had East Asian ancestry. This generated a right eye logarithm of minimal angle of resolution (logMAR) score, with negative numbers indicating ‘better than normal’ and positive numbers indicating ‘worse than normal’ distance vision.^34^ Scores ranged from −0.66 to +1.35.^35^ Phenome-wide association scans were performed using the PHEnome Scan ANalysis Tool (PHESANT), to find single nucleotide polymorphisms (SNPs) associated with a wide variety of traits in a hypothesis-free manner.^36,37^ We chose habitual right eye logMAR score because it incorporated correction of myopia (e.g. glasses), and non-receipt of corrective aids is a possible mechanism by which schizophrenia could cause poorer acuity.

We used the summary statistics for dental caries in the UK Biobank,^36^ established by ICD-10 diagnosis from healthcare records.^38^ There were 3646 cases and 416 885 controls with European ancestry (*N* = 420 531).

In a *post hoc* analysis, we also used binary myopia correction status, determined by reporting that glasses or contact lenses were needed mainly for short-sightedness,^39^ which yielded 33 358 cases and 386 580 controls of European ancestry (*N* = 419 938).

#### 23andme

23andme is a private company offering genotyping. Consenting customers were asked the following questions: ‘Have you ever been diagnosed by a doctor with near-sightedness (near objects are clear, far objects are blurry)?’ and ‘Are you near-sighted (near objects are clear, far objects are blurry)?’; and with the same descriptor, ‘What vision problems do you have?’ and ‘Prior to your LASIK eye surgery, what vision problems did you have?’. These questions identified 106 086 probable myopia cases and 85 757 controls used in subsequent meta-analysis (*N* = 191 841).^33^

#### Consortium of Refractive Error and Myopia

The Consortium of Refractive Error and Myopia (CREAM), designed to further knowledge of genetics of myopia and refractive error, comprised 34 079 participants aged ≥25 years who did not have major ocular conditions that could alter refractive error.^33^ All had refractive error measured and an average between the two eyes taken.^40^ Methods specific to each study within CREAM are described elsewhere.^41^ Linear regression was used to identify SNPs associated with spherical equivalent.^33^

#### The Genetic Epidemiology Research in Adult Health and Aging Cohort

The Genetic Epidemiology Research in Adult Health and Aging Cohort (GERA) cohort has been described in detail elsewhere.^42^ It is part of the Kaiser Permanente Research Program on Genes, Environment, and Health, and includes 34 998 adults who had spherical equivalent measured at least once between 2008 and 2014. Mean spherical equivalent from both eyes at first documented assessment was used in meta-analysis.^33^ Linear regression was used to determine SNPs associated with spherical equivalent.^33^

The above binary poor distance acuity status samples were combined in a meta-analysis with a *Z*-score method (*N* = 542 932).^33^

#### Meta-analysis of severe myopia in East Asian and South-East Asian ancestry participants

Meguro and colleagues performed a GWAS meta-analysis of 2549 patients with severe myopia and 11 547 healthy controls of East and South-East Asian ancestry, to identify SNPs associated with high (severe) myopia, which is expected to yield more genetic variants than milder forms.^43^ The studies variably defined high myopia as spherical equivalent in at least one eye of ≤−5.0, ≤−6.0 or ≤−9.0 dioptres, or having an axial length >26 mm. Controls without myopia were defined as spherical equivalent ≥−0.50 or ≥−1.0 dioptres in both eyes. Formal ophthalmic examination determined phenotypes.

### Instrument selection

All of our analyses used summary data. The PGC Schizophrenia Working group have identified 270 independent genetic loci across ancestries associated with schizophrenia as a binary phenotype with genome-wide significance.^7^ Around 60–80% of phenotype variance in schizophrenia can be attributed to genetic factors,^7^ and these genetic variants are estimated to account for 24% of this heritability.^7^ Fine-mapping has increased the credibility of many loci as containing causal genes, and genes preferentially expressed in brain tissues showed enriched associations.^7^

Across 542 934 individuals from the UK Biobank, 23andme, the GERA cohort and CREAM consortium, 449 genetic loci associated with myopia with genome-wide significance (*P* < 5 × 10^−8^) have been identified through meta-analysis.^33^ These analyses were restricted to participants of European ancestry. Refractive error has a heritability of 60–80%,^44^ and collectively, these SNPs are estimated to explain 18.4% of this heritability.^33^ The majority are in regions with known, plausible biological pathways to myopia and in genes preferentially expressed in ocular tissues.^33^

In the meta-analysis of East Asian and South-East Asian ancestry participants, nine genetic loci were discovered to be associated with high myopia with genome-wide significance.^43^

Where necessary, we calculated beta-values and standard errors for SNP associations with poor distance acuity from the meta-analysis *Z*-scores, using formulae in a supplement.

We applied the following criteria to identify instrument SNPs for each exposure: association with exposure in relevant ancestry sample at significance level *P* < 5 × 10^−8^; minor allele frequency (MAF) >0.005; imputation accuracy (only available for schizophrenia instruments) info score >0.7 and not in linkage disequilibrium with another instrument SNP (defined as correlation >0.2). We removed palindromic SNPs with MAF > 0.42 by using the *TwoSampleMR* package in R software (version 4.3.0 for Windows; Posit, Boston, MA, USA; https://posit.co/), because of potential uncertainty about which was the effect allele.^45,46^

When selecting SNPs associated with habitual logMAR score from the UK Biobank as instruments, we used the more lenient threshold of *P* < 5 × 10^−5^ because the number of variants associated at genome-wide significance was insufficient, and the same criteria for SNP selection otherwise.

### Mendelian randomisation analysis

We used instrumental SNPs identified as associated with the exposure in discovery samples as proxies for the exposure, and tested their associations with the outcome in the outcome sample, pooling these associations to create an overall effect estimate of the exposure on the outcome.

**Fig. 1:**
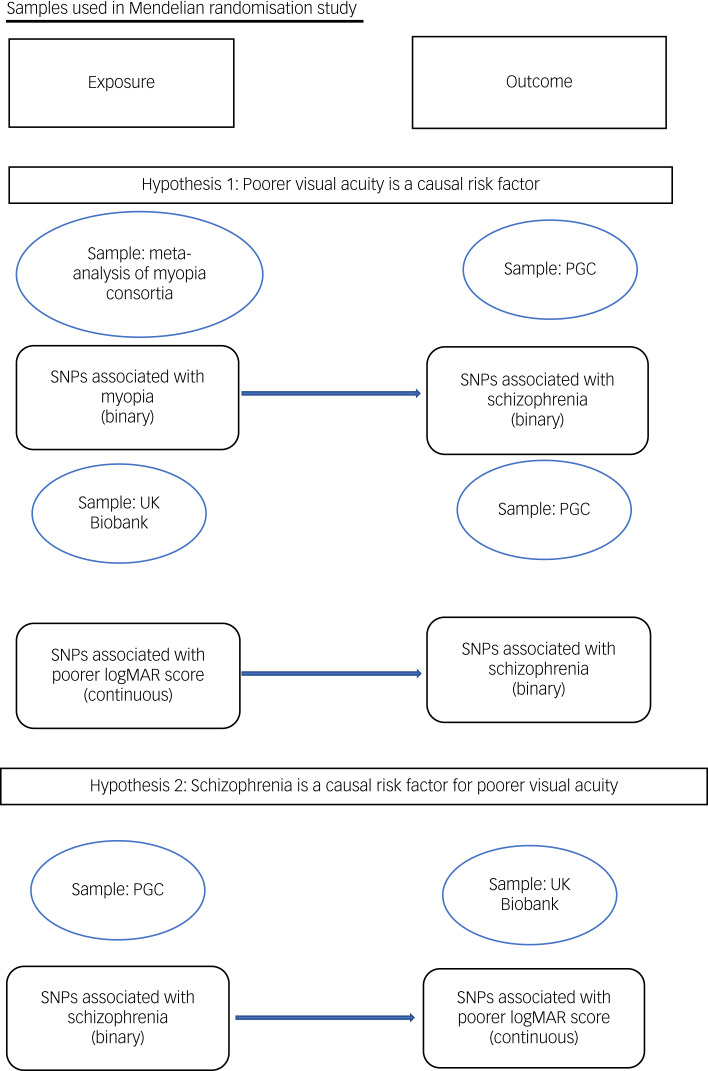
Samples used in Mendelian randomisation study. logMAR, logarithm of minimal angle of resolution; PGC, Psychiatric Genomics Consortium; SNP, single nucleotide polymorphism.

To test hypothesis 1 (that poor distance acuity is a causal risk factor for schizophrenia), we used SNPs identified in the meta-analysis of poor distance acuity samples as instrumental exposures, and tested their association with schizophrenia in the PGC from summary statistics. We also used SNPs associated with poorer habitual logMAR score measured at a distance of 4 m from the UK Biobank as instrumental exposures.^47^ To test hypothesis 2 (schizophrenia is a causal risk factor for poorer visual acuity), we used SNPs identified as associated with schizophrenia in the PGC as instrumental exposures, and tested their associations with poorer logMAR score in the UK Biobank (Fig. 1).

For our primary analyses, we used samples with European ancestry, as it was the group in which most relevant SNPs have been identified. Using statistics from homogenous ancestry groups reduces the chances of confounding by population structure and use of invalid instruments in Mendelian randomisation.^27,48^ We analysed data from samples with East Asian ancestry to test whether findings are consistent in other ancestry groups. No published schizophrenia GWAS that was sufficiently large was available for any other ancestry group.

We used the package *TwoSampleMR* to run analyses, using R version 4.0.3.^45,46,49^ Our primary analyses used the inverse variance-weighted (IVW) method, which pools SNP effect estimates weighted for the inverse variance of the ratio estimate.^50^ Where the outcome was binary (poor distance acuity or schizophrenia), we converted the association between exposure and outcome to an odds ratio.

### Sensitivity analyses

Mendelian randomisation methodology assumes absence of directional horizontal pleiotropy, where SNPs influence the outcome directly or via phenotypes other than the exposure, creating an artificial direction of effect.^51^ Therefore we also conducted Mendelian randomisation using several methods that are robust to violations: Mendelian randomisation-Egger, which does not constrain the intercept to zero; the weighted mode, which is valid if the largest group of SNPs are valid instruments; and the weighted median method, which is valid if >50% SNPs are valid instruments.^29^ We report the significance of the Mendelian randomisation-Egger intercept from a random-effects model to test for horizontal pleiotropy. As further sensitivity analyses, we conducted single SNP and ‘leave-one-out’ analysis, and the Mendelian randomisation pleiotropy residual sum and outlier (MR-PRESSO) test to identify outlying SNPs, with a significance threshold of 0.05.^52^ We performed Cochran's *Q*-test for heterogeneity, and generated scatter and funnel plots to visually inspect results. Symmetry of the funnel plot is evidence against directional pleiotropy.^53^

If a statistically significant association was found, we compared the results where poor distance acuity was the phenotypic exposure or outcome to dental caries, to see if overall neglect of physical healthcare was a likely mechanism by which the association could occur.

### Schizophrenia as a cause of myopia non-correction

As a *post hoc* analysis, we also tested whether schizophrenia as exposure was causally associated with reporting or not reporting needing glasses for short sight as an outcome.

## Results

### Hypothesis 1: poor distance acuity is a causal risk factor for schizophrenia in European ancestry samples

**Fig. 2:**
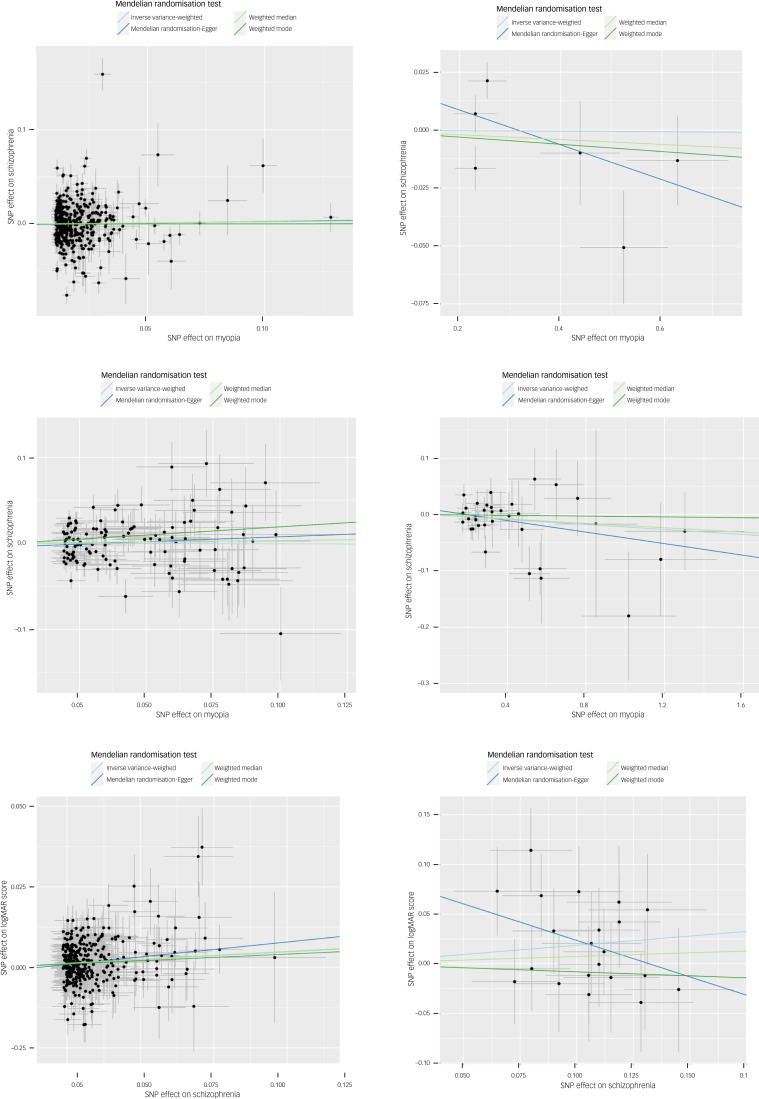
Graphical results of Mendelian randomisation. logMAR, logarithm of minimal angle of resolution; SNP, single nucleotide polymorphism.

We found no evidence of a causal effect of poor distance acuity on schizophrenia (odds ratio 1.00, 95% CI 0.91–1.10; *P* = 0.955) (Table 1, Fig. 2 and Supplementary Figs 1–3).

**Table 1:** Results of Mendelian randomisation analysis

Mendelian randomisation method	Number of SNPs	Beta coefficient [95% CI]	s.e.	Odds ratio [95% CI]	*P*-value
Poor distance acuity as exposure, schizophrenia as outcome in European ancestry sample					
Inverse variance-weighted	405	0.003 [−0.090 to 0.096]	0.048	1.003 [0.913–1.101]	0.955
Mendelian randomisation-Egger	405	0.033 [−0.177 to 0.243]	0.107	1.034 [0.838–1.275]	0.757
Weighted median	405	0.001 [−0.124 to 0.126]	0.064	1.001 [0.883–1.135]	0.985
Weighted mode	405	0.023 [−0.070 to 0.117]	0.048	1.024 [0.932–1.124]	0.625
Severe myopia as exposure, schizophrenia as outcome in East Asian ancestry sample					
Inverse variance-weighted	6	−0.001 [−0.054 to 0.052]	0.027	0.999 [0.947–1.053]	0.962
Mendelian randomisation-Egger	6	−0.075 [−0.222 to 0.072]	0.075	0.928 [0.801–1.074]	0.373
Weighted median	6	−0.010 [−0.055 to 0.034]	0.023	0.990 [0.947–1.035]	0.648
Weighted mode	6	−0.015 [−0.096 to 0.065]	0.041	0.985 [0.909–1.067]	0.721
Poorer habitual visual acuity as exposure, schizophrenia as outcome in European ancestry sample					
Inverse variance-weighted	117	−0.006 [−0.125 to 0.114]	0.061	0.994 [0.882–1.121]	0.927
Mendelian randomisation-Egger	117	0.114 [−0.181 to 0.410]	0.151	1.121 [0.834–1.507]	0.450
Weighted median	117	0.032 [−0.105 to 0.169]	0.070	1.033 [0.900–1.184]	0.647
Weighted mode	117	0.191 [−0.239 to 0.621]	0.219	1.210 [0.787–1.861]	0.386
Poorer habitual visual acuity as exposure, schizophrenia as outcome in East Asian ancestry sample					
Inverse variance-weighted	34	−0.022 [−0.055 to 0.010]	0.017	0.976 [0.947–1.010]	0.179
Mendelian randomisation-Egger	34	−0.051 [−0.124 to 0.021]	0.037	0.950 [0.883–1.022]	0.176
Weighted median	34	−0.020 [−0.068 to 0.029]	0.025	0.980 [0.934–1.029]	0.426
Weighted mode	34	−0.004 [−0.088 to 0.081]	0.043	0.996 [0.916–1.084]	0.933
Schizophrenia as exposure, poorer habitual visual acuity (logMAR score) as outcome in European ancestry sample					
Inverse variance-weighted	355	0.024 [0.014–0.033]	0.005	−	<0.001
Mendelian randomisation-Egger	355	0.047 [0.007–0.087]	0.022	−	0.022
Weighted median	355	0.025 [0.012–0.038]	0.007	−	<0.001
Weighted mode	355	0.020 [−0.017 to 0.058]	0.019	−	0.290
Schizophrenia as exposure, poorer habitual visual acuity (logMAR) score as outcome in East Asian ancestry sample					
Inverse variance-weighted	21	0.186 [−0.008 to 0.379]	0.099	−	0.060
Mendelian randomisation-Egger	21	−0.735 [−0.174 to 0.277]	0.516	−	0.171
Weighted median	21	0.0732 [−0.182 to 0.328]	0.130	−	0.574
Weighted mode	21	−0.081 [−0.533 to 0.371]	0.231	−	0.729
Schizophrenia as exposure, glasses use for myopia as outcome in European ancestry sample					
Inverse variance-weighted	221	−0.055 [−0.084 to −0.026]	0.015	0.947 [0.920–0.974]	<0.001
Mendelian randomisation-Egger	221	−0.239 [−0.353 to −0.125]	0.058	0.787 [0.703–0.882]	<0.001
Weighted median	221	−0.094 [−0.127 to −0.062]	0.017	0.910 [0.880–0.940]	<0.001
Weighted mode	221	−0.144 [−0.222 to −0.066]	0.040	0.866 [0.801–0.936]	<0.001

SNP, single nucleotide polymorphism; logMAR, logarithm of minimal angle of resolution.

### Hypothesis 1: severe myopia is a causal risk factor for schizophrenia in East Asian ancestry samples

There was also no evidence of association between severe myopia as exposure and schizophrenia as outcome in East Asian ancestry samples (odds ratio 1.00, 95% CI 0.95–1.05; *P* = 0.962) (Table 1, Fig. 2 and Supplementary Figs 1–3).

### Hypothesis 1: poorer habitual visual acuity is a risk factor for schizophrenia in European ancestry samples

Consistent with our previous analyses, using poorer continuous logMAR score as the exposure phenotype to represent visual impairment in a sample of people with European ancestry found no evidence of an association with schizophrenia as outcome (odds ratio for one-point poorer logMAR score: 0.99, 95% CI 0.88–1.12; *P* = 0.927) (Table 1, Fig. 2 and Supplementary Figs 1–3).

### Hypothesis 1: poorer habitual visual acuity is a risk factor for schizophrenia in East Asian ancestry samples

We similarly found no association between poorer visual acuity and schizophrenia in East Asian ancestry samples in the IVW analysis (odds ratio per one-point poorer logMAR score: 0.98, 95% CI 0.94–1.01; *P* = 0.179) or any other method.

Throughout hypothesis 1 analyses, sensitivity analyses were in keeping with primary analyses (Table 1, Fig. 2 and Supplementary Figs 1–3).

### Hypothesis 2: schizophrenia is a causal risk factor for poorer habitual visual acuity in European ancestry samples

We found evidence of a causal effect based on schizophrenia instruments’ association with the outcome (poorer habitual visual acuity) when using the IVW method (beta coefficient: 0.024, 95% CI 0.014–0.033; *P* = 9.63 × 10^−7^) (Table 1, Fig. 2 and Supplementary Figs 1–3). The direction of effect was consistent across other Mendelian randomisation methods. The Mendelian randomisation-Egger intercept indicated no evidence of pleiotropy (*P* = 0.877), suggesting a true effect, but Cochran's *Q*-statistic showed evidence of heterogeneity (*P* = 0.029). However, the funnel plot was broadly symmetrical (Supplementary Fig. 3(e)). MR-PRESSO did not identify outlying SNPs, and IVW results remained significant in single SNP and leave-one-out analyses, suggesting outliers were not responsible for the association (Supplementary Figs 1(e) and 2(e)).

### Hypothesis 2: schizophrenia is a causal risk factor for poorer habitual visual acuity in East Asian ancestry samples

The causal estimate for East Asian ancestry samples was larger (beta coefficient: 0.186, 95% CI −0.008 to 0.379; *P* = 0.060) and directionally consistent with the estimate for European ancestry. However, it did not reach statistical significance, and the results from this analysis were imprecise (Table 1, Fig. 2 and Supplementary Figs 1–3).

### *Post hoc* analysis: schizophrenia is a causal risk factor for myopia

Schizophrenia was negatively associated with reporting glasses use for myopia (odds ratio 0.95, 95% CI 0.920–0.974; *P* = 0.0002). Sensitivity methods were consistent with this (Table 1, Fig. 2 and Supplementary Figs 1–3).

### Schizophrenia as exposure and dental caries as outcome in European ancestry samples

There was a negative association between schizophrenia as exposure and dental caries as outcome when using identical methods to those used to test hypothesis 2 (odds ratio 0.948, 95% CI 0.903–0.995; *P* = 0.032) (Table 1, Fig. 2, Supplementary Figs 1–3 and Supplementary Table 1).

## Discussion

### Main findings

We found no evidence to support a causal role of poor distance acuity in the development of schizophrenia. Conversely, we found evidence that schizophrenia is a casual risk factor for poorer visual acuity in people of European ancestry. Although Cochran's *Q*-test showed evidence of heterogeneity, this test has been suggested to be oversensitive with large sample sizes.^54^ There was little evidence of heterogeneity between results from European and East Asian ancestry groups, and the smaller sample size resulted in less precise estimates for the analyses in participants with East Asian ancestry. The size of the effect was very small: less than a one-line difference on the visual acuity chart.

In our comparison analysis to investigate neglect of physical healthcare as a possible mechanism, we found a negative association when dental caries was the outcome. This is likely to be because people with schizophrenia were less likely to receive dental treatment and have this outcome recorded, in keeping with neglect of healthcare. Our finding that schizophrenia was negatively associated with reporting glasses use for myopia further suggests that neglect of eyecare specifically may be a mechanism.

### Strengths and limitations

We tested the effect of poor distance acuity on schizophrenia risk and schizophrenia on risk of poor distance acuity, using a methodology not previously applied to this question. We were able to exclude reverse causation and reduce confounding from unobserved environmental variables. The SNPs from European ancestry samples were credible instruments because of their strong associations with the exposures in meta-analyses and replication samples and recognised biological pathways to the exposures.

There are, however, limitations to our methodology. Mendelian randomisation relies on the assumption of no directional horizontal pleiotropy, which cannot be proven, although we have carried out multiple sensitivity analyses to detect this.^52^ Sample overlap can bias results toward the null in two-sample Mendelian randomisation.^55^ We have aimed to exclude this where possible, by using international rather than UK samples alongside the UK Biobank. Also, as schizophrenia is a relatively rare condition, it is unlikely that many cases would be present in the other studies. We cannot exclude the possibility of collider bias as a possible explanation for the finding that schizophrenia contributes causally to myopia.^56^ However, myopia did not, to our knowledge, influence the chance of selection, making this unlikely.

There are also limitations regarding choice of phenotypes. The studies used to detect genetic variants associated with poor distance acuity used different phenotypic measures, including self-reported rather than objective measures. However, self-reported short-sightedness has been shown to be a reliable indicator of myopia.^57^ Perhaps more importantly, we were unable to account for correction of vision using aids when poor distance acuity was the exposure, and so cannot exclude modification of risk through this. There is some evidence that correction of myopia reduces the association with subsequent schizophrenia.^12^ Indeed, people self-reporting myopia may be more likely to be using aids than people who are unaware of their poor eyesight. We had no information regarding age at onset of myopia, which would alter the dose of exposure received and whether it was received during central nervous system development, which may modify associations, particularly as schizophrenia is a neurodevelopmental disorder.^58^

Further, we have used techniques that assume a linear relationship between exposure and outcome. As most cases of myopia lead to mild, rather than severe, distance visual acuity impairment, we consider the linear relationship likely despite the non-linear shape of the PaSZ model across degrees of visual impairment. We were nevertheless restricted to using severe myopia as the phenotype in one analysis of East Asian ancestry samples. However, the authors of this GWAS used this phenotype to yield more variants associated with myopia overall.^43^ Our results are not necessarily inconsistent with more severe forms of visual impairment, such as retinal conditions with different genetic determinants, being a causal risk factor for psychotic illnesses.

### Comparison with other studies

We are unaware of any prior Mendelian randomisation studies on this topic. Three longitudinal studies of children report a positive association between ocular dysfunction and subsequent psychotic symptoms or diagnoses.^15,59,60^ However, similar studies of adolescents and adults give mixed findings, with some reporting no association, some a positive association and some a negative association.^12,61–64^ There are, therefore, two likely explanations for our null result when visual impairment is the exposure. The first is that that myopia or mild visual impairment is a causal risk factor for psychosis, but only during a critical period of central nervous system development; this was captured in the studies of children, but not with our phenotype measure in this study, because genetic variants for childhood visual impairment specifically may not fully overlap with our instrument, which related to lifelong phenotype.^40^ The second is that there is truly no causal effect of poor distance acuity on schizophrenia, and the finding in other studies is a result of residual confounding. We consider this the most likely because there was no trend toward a positive association in our study, which might be expected if poor distance acuity was relevant at any time point. Further, psychotic symptoms, as measured in some of the studies, do not equate to having schizophrenia and might be non-pathological or result from other conditions associated with poorer eyesight, such as depression.^65^

In conclusion, our results suggest that mild visual acuity impairment is not causally associated with subsequent schizophrenia, but schizophrenia as an exposure is causally associated with poorer visual acuity, although the effect was very small. Regardless of the mechanism, this study highlights a potentially unmet health need in people with schizophrenia. A combination of visual side-effects from medications, and possible lower propensity to seek treatment, could be potentially important areas to investigate to improve quality of care in this group. Shared actions of genes may also account for some of the association. With further research, this may translate into clinical benefit for patients, such as wider use of routine eye tests at the annual physical health check for people with serious mental illnesses. Future research could aim to use mediation analyses to establish mechanisms by which schizophrenia might lead to poorer vision, and to assess whether interventions to improve eye health for people with schizophrenia are acceptable and effective.

## Data Availability

Data used in this study are publicly available. Data can be accessed from the Psychiatric Genomics Consortium (https://www.med.unc.edu/pgc/), UK Biobank (http://www.nealelab.is/uk-biobank) and papers referenced in the article.
