# Investigating the association between schizophrenia and distance visual acuity: Mendelian randomisation study – CORRIGENDUM

**DOI:** 10.1192/bjo.2023.556

**Published:** 2024-04-02

**Authors:** Natalie Shoham, Diana Dunca, Claudia Cooper, Joseph F. Hayes, Andrew McQuillin, Nick Bass, Gemma Lewis, Karoline Kuchenbaecker

**Keywords:** Psychiatric epidemiology, visual impairment, schizophrenia, psychosis, Mendelian randomisation, corrigendum

The original discussion read that sample overlap can bias results towards the null in two sample MR. This should read: Use of weak instruments can bias results towards the null in two sample MR, except possibly in the presence of sample overlap.^1,2^ The authors apologise for this oversight.

In two sample MR analysis, when the outcome is continuous, the use of weak instruments could bias the results towards the observational association and could inflate the type I error. The degree of bias and Type I inflation depend on several factors, including the magnitude of the true causal effect, the degree of confounding between the risk factors and the outcome, as well as on the degree of overlap between the two samples.^1,2^ If the two samples are independent or almost independent (as we anticipate in this study) and the true causal effect not zero, the bias might be in the direction of the null hypothesis.
